# Left Ventricular Venting during On-Pump Beating-Heart Coronary Artery Bypass Grafting for Advanced Ischemic Cardiomyopathy: A Surgical Strategy to Achieve Complete Revascularization

**DOI:** 10.5761/atcs.nm.26-00080

**Published:** 2026-07-01

**Authors:** Tomo Yoshizumi, Tomonari Uemura, Yasunari Hayashi, Sachie Terazawa, Yoshiyuki Tokuda, Yuji Narita, Masato Mutsuga

**Affiliations:** Department of Cardiac Surgery, Nagoya University Graduate School of Medicine, 65 Tsurumai-cho, Showa-ku, Nagoya, Aichi, Japan

**Keywords:** coronary artery bypass graft, ischemic cardiomyopathy, advanced heart failure, on-pump beating-heart coronary artery bypass graft

## Abstract

We describe a surgical strategy combining on-pump beating-heart coronary artery bypass grafting (OPBH CABG) with left ventricular (LV) venting for patients with advanced ischemic cardiomyopathy (ICM) with severe LV dysfunction and marked LV dilation. This strategy provides LV decompression, reduces myocardial workload, and improves surgical exposure during coronary anastomosis, facilitating complete revascularization. Between January 2013 and September 2024, 7 patients underwent OPBH CABG with LV venting. Six patients had chronic ICM with dilated ventricles and severe systolic dysfunction, and 1 presented with acute coronary syndrome and impaired LV function. The median age was 65 years (interquartile range [IQR], 61–80). The median left ventricular ejection fraction was 21.7% (IQR, 19.2–27.9), and the median left ventricular end-diastolic diameter was 61.6 mm (IQR, 58.7–69.3). Complete revascularization was achieved in all patients without additional mechanical circulatory support. There were no in-hospital deaths or postoperative complications. This strategy may enable complete revascularization in selected high-risk patients while contributing to myocardial unloading.

## Introduction

Coronary artery bypass grafting (CABG) improves survival in patients with ischemic cardiomyopathy (ICM) and reduced left ventricular (LV) function.^1)^ However, CABG in patients with severe LV dysfunction accompanied by LV dilation is associated with a higher risk of postoperative complications, including postcardiotomy cardiogenic shock, and the optimal surgical strategy remains controversial.

Off-pump CABG may reduce perioperative complications, but intraoperative cardiac displacement can cause hemodynamic instability and may require conversion to on-pump surgery.^2)^ Unplanned conversion from off-pump to on-pump CABG has been reported to be associated with markedly increased operative mortality. In addition, off-pump CABG may result in fewer distal anastomoses.

On-pump beating-heart CABG (OPBH CABG) maintains circulatory support without cardioplegic arrest. Although some studies have reported fewer perioperative complications compared with conventional on-pump arrested-heart CABG (OPAH CABG), other reports have suggested a higher incidence of irreversible myocardial ischemia, leaving its safety and efficacy controversial.^3,4)^

Recent expert consensus documents have highlighted the potential benefits of perioperative temporary mechanical circulatory support (MCS).^5)^ However, concerns remain regarding device-related complications and the complexity of MCS management.

In this study, we present a surgical strategy using OPBH CABG with LV venting for patients with advanced ICM with severe LV dysfunction and LV dilation. LV venting provides LV decompression, reduces myocardial workload, and improves surgical exposure during coronary anastomosis, thereby enabling complete surgical revascularization.

## Case Report

### Surgical technique

OPBH CABG with LV venting was considered for patients with acute coronary syndrome (ACS) accompanied by severe LV dysfunction, as well as for those with ICM characterized by reduced contractility and LV dilation.

Cardiopulmonary bypass (CPB) was established with arterial inflow via the ascending aorta and venous drainage from the right atrium using a 2-stage venous cannula. After initiation of CPB, LV venting was performed through the right upper pulmonary vein using a standard vent catheter.

Once full-flow CPB was established, mechanical ventilation was withheld throughout the procedure to minimize the risk of air entrainment into the left heart system via the LV vent. In addition, careful attention was paid to maintaining adequate intracardiac filling and avoiding excessive negative pressure during suction. Routine use of an aortic root vent was not considered necessary.

Continuous suction was applied to maintain LV decompression, and the suction level was manually adjusted by the surgeon in collaboration with the perfusionist based on tactile assessment of LV distension and wall tension, particularly during cardiac repositioning. During CPB, mean systemic arterial pressure was managed according to standard practice, with adjustments based on patient-specific risks, including cerebrovascular considerations. Vent suction was generally adjusted to reduce arterial pulsatility to a near-pulseless level while maintaining adequate systemic perfusion. Excessive negative pressure may cause myocardial injury or distort LV geometry, potentially compromising surgical exposure and coronary anastomosis; therefore, the suction level was carefully titrated to achieve adequate LV unloading without ventricular collapse.

Revascularization was actively pursued even in technically challenging coronary territories with limited exposure due to marked LV dilation. Particular attention was directed to the posterolateral LV wall, including the obtuse marginal, posterolateral, and atrioventricular nodal branches, to achieve complete revascularization. Distal coronary anastomoses were performed using standard beating-heart techniques. Proximal anastomoses of free grafts to the ascending aorta were constructed using a proximal anastomotic device.

### Clinical experience

Seven patients underwent OPBH CABG with LV venting. Preoperative patient characteristics are summarized in **Table 1**. The median age was 65 years (interquartile range [IQR], 61–80). Emergent or urgent surgery was performed in 3 patients (42.9%). Preoperative temporary MCS was required in 3 patients (42.9%), all of whom were supported with an intra-aortic balloon pump (IABP). The median preoperative EuroSCORE II was 9.6% (IQR, 5.36–21.4).

**Table 1:** Preoperative patient characteristics

Patient no,	Age (years)	Sex	Clinical diagnosis	NYHA class	Preoperative MCS	Urgency	Preoperative LVEF (%)	Preoperative LVEDD (mm)	EuroSCORE II (%)
1	62	Male	AMI due to LMCA disease	IV	IABP	Emergent	20	N/A	32.8
2	59	Male	Chronic ICM	IV	IABP	Urgent	12.1	86.3	9.60
3	87	Female	Chronic ICM	IV	IABP	Elective	21.7	53.4	10.1
4	65	Male	Chronic ICM	III	None	Elective	28.3	71.4	1.92
5	83	Male	Chronic ICM	II	None	Elective	28.3	60.2	5.29
6	50	Male	Chronic ICM	II	None	Elective	18.4	58.2	5.41
7	77	Male	Chronic ICM	IV	IABP	Urgent	27.4	63	34.9

AMI, acute myocardial infarction; LMCA, left main coronary artery; LVEF, left ventricular ejection fraction; LVEDD, left ventricular end-diastolic diameter; MCS, mechanical circulatory support; NYHA, New York Heart Association; ICM, ischemic cardiomyopathy

The median preoperative left ventricular ejection fraction (LVEF) was 21.7% (IQR, 19.2–27.9), and the median left ventricular end-diastolic diameter (LVEDD) was 61.6 mm (IQR, 58.7–69.3).

Operative data are summarized in **Table 2**. The median CPB time was 182 min (IQR, 113–205). The median number of distal anastomoses was 3 (IQR, 3–4). Complete revascularization was achieved in all patients, and no patient required newly initiated temporary MCS during or after surgery.

**Table 2:** Intraoperative variables

Patient no.	Graft configuration	Operation time (min)	CPB time (min)	Number of distal anastomoses	Complete revascularization	Additional MCS	Conversion to arrested-heart CABG
1	LITA-LAD, SVG-OM, SVG-RI	462	247	3	Yes	None	None
2	LITA-LAD, SVG-RI-PL	390	205	3	Yes	None	None
3	LITA-LAD, SVG-PDA-AV	298	139	3	Yes	None	None
4	RITA-LAD, LITA-PL, SVG-PDA	290	113	3	Yes	None	None
5	RITA-LAD, LITA-RI, SVG-PDA	280	107	3	Yes	None	None
6	LITA-LAD, SVG-PDA, free RITA-Diag-PL	406	182	4	Yes	None	None
7	RITA-LAD, SVG-PDA, SVG-PL, SVG-Diag	434	187	4	Yes	None	None

LITA, left internal thoracic artery; RITA, right internal thoracic artery; SVG, saphenous vein graft; LAD, left anterior descending artery; OM, obtuse marginal branch; RI, ramus intermedius; PL, posterolateral branch; PDA, posterior descending artery; AV, atrioventricular branch; Diag, diagonal branch; CPB, cardiopulmonary bypass; MCS, mechanical circulatory support; CABG, coronary artery bypass grafting

Early postoperative outcomes are presented in **Table 3**. Overall, postoperative complications were infrequent, with no in-hospital deaths, re-exploration for bleeding, or central nervous system complications.

**Table 3:** Postoperative outcomes

Patient no.	In-hospital death	Postcardiotomy cardiogenic shock	Perioperative MI	Stroke	Reoperation for bleeding	Renal failure requiring dialysis	Prolonged ventilation (>72 h)	ICU stay (days)	Deep sternal wound infection	Postoperative hospital stay (days)	Early graft patency (%)
1	None	None	None*	None	None	None	Yes	10	None	30	100
2	None	None	None	None	None	None	None	7	None	91**	100
3	None	None	None	None	None	None	None	11	None	42	100
4	None	None	None	None	None	None	None	3	None	16	100
5	None	None	None	None	None	None	None	5	None	22	100
6	None	None	None	None	None	None	None	3	None	15	75***
7	None	None	None	None	None	None	Yes	11	None	27	100

*Emergency surgery for preoperative acute MI; no evidence of new perioperative myocardial infarction.

**Preoperative IABP was removed postoperatively; however, a durable LVAD was implanted on postoperative day 91 due to persistent left ventricular dysfunction.

***The PL anastomosis of the sequential free RITA graft was occluded; all other grafts remained patent.

MI, myocardial infarction; IABP, intra-aortic balloon pump; LVAD, left ventricular assist device; PL, posterolateral branch; RITA, right internal thoracic artery; ICU, intensive care unit

Perioperative myocardial infarction occurred in 1 patient. This patient had preoperative acute myocardial infarction caused by thrombotic occlusion of the left main trunk and required IABP support. Emergency percutaneous coronary intervention was unsuccessful, and CABG was subsequently performed. The patient was successfully weaned from IABP and discharged ambulatory. In the remaining patients, no significant creatine kinase-myocardial band elevation, new Q waves on electrocardiography, or new regional wall motion abnormalities were observed.

Another patient with chronic ICM, who had a preoperative LVEF of 12.1% and an LVEDD of 86.3 mm and required preoperative IABP support, was successfully weaned from IABP postoperatively; however, persistent LV dysfunction necessitated durable left ventricular assist device (LVAD) implantation 91 days after surgery.

Postoperative graft evaluation was performed in all patients. Only 1 sequential anastomosis using a free internal thoracic artery graft to the left circumflex territory was occluded, resulting in an early graft patency rate of 95.7% (22/23).

## Discussion

In this study, we describe a reproducible surgical strategy combining OPBH CABG with LV venting in patients with advanced ICM. In such patients, marked LV dilation often makes exposure of the lateral and posterolateral coronary territories technically challenging during surgical revascularization.

LV venting provides ventricular decompression and improves operative exposure of the target coronary arteries during distal anastomosis. By providing ventricular decompression and improved operative exposure, this approach facilitates complete revascularization and may contribute to favorable early postoperative outcomes. Conceptually, this strategy may contribute to ventricular unloading without requiring additional device implantation.

According to the *2021 American Association for Thoracic Surgery (AATS) Expert Consensus Document: Coronary Artery Bypass Grafting in Patients with Ischemic Cardiomyopathy and Heart Failure*,^5)^ LVEF <25% and severe LV dilatation are considered high-risk features, and such patients require thorough preoperative multidisciplinary assessment, including optimization with temporary MCS or evaluation for candidacy for durable LVAD therapy. However, the document does not clearly address the optimal use of CPB during the intraoperative period.^5)^

A national database analysis reported that unplanned conversion from off-pump CABG occurred in 6.1% of cases and was associated with an operative mortality of 12.5%.^2)^ These findings suggest that a planned on-pump strategy may be reasonable in patients with severely reduced LVEF.

Several meta-analyses have compared clinical outcomes between OPBH CABG and conventional OPAH CABG. Although OPBH CABG has been associated with lower early morbidity and mortality than OPAH CABG, it has also been suggested that OPBH CABG may be associated with a lower number of distal anastomoses and a lower rate of complete revascularization.^3,6)^

In contrast, the present case series demonstrated a high rate of complete revascularization despite the inclusion of patients with ICM and a markedly dilated LV. In OPBH CABG without LV venting, obtaining an adequate surgical field for anastomosis in the left circumflex territory can be challenging because of limited intrapericardial space due to LV dilation. Therefore, the good surgical exposure achieved by LV venting may be one of the key factors contributing to the high complete revascularization rate observed in the present series.

Previous studies have suggested that OPBH CABG may be associated with irreversible myocardial injury caused by insufficient distal perfusion during beating-heart revascularization.^5)^ In contrast, no evidence of new myocardial injury was observed in the present series. This favorable outcome may be related to effective intraoperative LV decompression, which may reduce myocardial oxygen demand and mitigate ischemic vulnerability.

Temporary MCS, such as Impella, has been reported to improve early outcomes in high-risk CABG by providing stable hemodynamic support.^7)^ However, its use may be associated with device-related complications, the need for anticoagulation, and increased costs.

In contrast, OPBH CABG with LV venting in the present series required no additional MCS and was associated with favorable perioperative outcomes. These findings suggest that OPBH CABG with LV venting may represent a feasible revascularization strategy for patients with advanced ICM, allowing stable intraoperative hemodynamics and adequate surgical exposure while facilitating complete revascularization.

### Study limitations

This study has several limitations. It was a retrospective, single-center study with a small sample size and no control group. In addition, the choice of surgical technique was influenced by individual surgeon preference and was not standardized. The patient population was heterogeneous, including 1 patient with ACS. However, the remaining patients had chronic ICM with severe LV dysfunction and dilation, which represents the primary target population of this surgical strategy. In the patient with ACS, LV venting was applied based on the same rationale of reducing intraoperative LV workload and was considered conceptually applicable.

Nevertheless, the favorable outcomes observed in this series suggest that OPBH CABG with LV venting may represent a feasible strategy for achieving complete revascularization without the need for additional temporary MCS and with a low incidence of early postoperative complications in selected high-risk CABG patients.

## Conclusion

OPBH CABG with LV venting may represent a feasible surgical strategy for patients with advanced ICM with severely reduced LVEF and LV dilation. By providing ventricular decompression and improved operative exposure, this approach facilitates complete revascularization while minimizing myocardial injury during surgical manipulation, contributing to favorable early postoperative outcomes.

## Declarations

### Data availability

The datasets used and/or analyzed during the current study are available from the corresponding author upon reasonable request.
